# Design of High-Gain Antenna Arrays for Terahertz Applications

**DOI:** 10.3390/mi15030407

**Published:** 2024-03-18

**Authors:** Xinran Ji, Yu Chen, Jing Li, Dian Wang, Yue Zhao, Qiannan Wu, Mengwei Li

**Affiliations:** 1 School of Instrument and Electronics, North University of China, Taiyuan 030051, China; jixinran@sxgkd.edu.cn (X.J.);; 2 School of Information Engineering, Shanxi Vocational University of Engineering Science and Technology, Jinzhong 030619, China; 3 School of Instrument and Intelligent Future Technology, North University of China, Taiyuan 030051, China; 4 Academy for Advanced Interdisciplinary Research, North University of China, Taiyuan 030051, China; 5 Center for Microsystem Integration, North University of China, Taiyuan 030051, China; 6 School of Semiconductors and Physics, North University of China, Taiyuan 030051, China; 7 Key Laboratory of Dynamic Measurement Technology, North University of China, Taiyuan 030051, China

**Keywords:** terahertz antenna, GCPW, high gain, VSWR, SIW

## Abstract

A terahertz band (0.1–10 THz) has the characteristics of rich spectrum resources, high transmission speed, strong penetration, and clear directionality. However, the terahertz signal will suffer serious attenuation and absorption during transmission. Therefore, a terahertz antenna with high gain, high efficiency, and wide bandwidth is an indispensable key component of terahertz wireless systems and has become a research hotspot in the field of antennas. In this paper, a high-gain broadband antenna is presented for terahertz applications. The antenna is a three-layer structure, fed by a grounded coplanar waveguide (GCPW), using polytetrafluoroethylene (PTFE) material as the dielectric substrate, and the metal through-hole of the dielectric substrate forms a substrate-integrated waveguide (SIW) structure. The metal fence structure is introduced to reduce the coupling effect between the radiation patches and increase the radiation bandwidth and gain. The center frequency is 0.6366 THz, the operating bandwidth is 0.61–0.68 THz, the minimum value of the voltage standing wave ratio (VSWR) is 1.00158, and the peak gain is 13.14 dBi. In addition, the performance of the designed antenna with a different isolation structure, the length of the connection line, the height of the substrate, the radius of the through-hole, and the thickness of the patch is also studied.

## 1. Introduction

With the continuous development of communication technology, people’s demand for communication speed is becoming increasingly urgent. Terahertz (THz) communication technology, as an emerging technology, can meet people’s growing demand for communication. THz technology is a very important frontier cross field. In the early stage, due to the performance of THz-related equipment and instruments, the development of THz communication systems was slow, and there were few studies on THz antennas. In any communication system, the performance of the antenna is crucial. In recent years, with the development of THz technology and related devices, the development of THz communication systems has been very rapid, and higher requirements have been put forward for THz antennas. The THz band is between the millimeter wave and the infrared band in the electromagnetic spectrum, including an extremely wide frequency range from 0.1 THz to 10 THz, and the corresponding wavelength is 30 μm to 3 mm. The low-frequency part of the THz wave intersects the millimeter wave, and the high-frequency part intersects the infrared wave, making the THz technology a cutting-edge technology in electronics and photonics [1]. THz electromagnetic waves have the merits of high penetration, low penetration loss, and high resolution. The rich spectrum resources can easily support high-speed wireless transmission up to 100 Gbps, so this frequency band is considered to be a potential working frequency band for next-generation wireless communications. In addition, this frequency range also has some other properties, such as the ability to penetrate optically opaque materials such as clothing, paper, cardboard, and many plastics or ceramics. For THz imaging, hidden objects such as weapons and knives in parcels, clothes, or mattresses can be detected.

A THz antenna is an indispensable part of THz wave detection. The performance of a THz antenna directly affects the quality of the whole detection system. Among the parameters of the antenna, the working bandwidth and gain of the antenna have a great influence on the responsivity of the THz detector [2]. At present, there are still many shortcomings in the research of THz antennas. The THz antenna works in the high-frequency band, and the size of the antenna is greatly reduced compared with conventional antennas such as microwave antennas. Compared with microwave antennas, most THz antennas have higher energy loss and lower manufacturing accuracy. Therefore, improving the performance of THz detectors is a huge challenge. Coupling more energy is a suitable solution to improve the performance of the detector.

Since THz waves are easily absorbed by substances such as water molecules or oxygen molecules, air attenuation is the main problem of THz wave propagation, and high-gain antennas are one of the effective ways to solve this problem [3,4]. At present, there are four main propagation windows in the THz band of 0.1–1 THz, which are [0.38–0.44 THz], [0.45–0.52 THz], [0.62–0.72 THz], and [0.77–0.92 THz] [5]. In 2015, Kanaya [6] designed a 1 × 4-slot dipole antenna array on an indium phosphide substrate. The radiating element was designed on a gold metal (thickness: 1 μm) and excited by a coplanar waveguide (CPW). The gain of the antenna was 7.35 dBi at a resonant frequency of 0.3 THz. In 2018, Kushwaha [7] proposed a design for a high-gain THz microstrip patch antenna based on photonic crystals, using a polyimide substrate, and the gain was 7.934 dB and the resonant frequency was 0.6308 THz. In 2019, to further improve the gain, Nurfitri [8] proposed a design for a 1 × 3-series-fed antenna array on a Rogers RT duroid 5880 dielectric substrate, achieving a gain of 11.2 dBi at 0.312 THz. In 2020, in order to achieve the miniaturization and reconfiguration of the THz antenna, Abohmra [9] designed an antenna with a polyethylene nathoate substrate, operating in the frequency band of 0.9–1.2 THz. The radiation unit contained a gold and perovskite (methyllead iodide) hybrid material with a size of 70 × 119 μm^2^. The antenna has a gain of 11.3 dBi and a radiation efficiency of 83%. For THz imaging and THz communication applications, Hlali [10] designed a reconfigurable non-reciprocal antenna array based on magnetized graphene at 1.48–2.67 THz. The chemical potential (μc) and magnetic field (B) of graphene were obtained by using the iterative process method of the wave concept. Silicon (Si) complementary metal oxide semiconductor (CMOS) technology plays a vital role in low-cost THz integrated circuits due to its low cost and ease of integration in both analog and digital forms. The THz antenna can also be integrated into the silicon liner. Lee C [11] designed a 2 × 2 THz antenna array on a CMOS chip using the working frequency band of 1.48–2.67 THz and the CMOS process. The gain measured by the antenna array was 8.9 dBi. In 2021, in the operating frequency band of 0.194–0.196 THz, Alibakhshikenari [12] fabricated a 2 × 3 graphene-based antenna array on a polyimide substrate. Metamaterial and substrate-integrated waveguide (SIW) techniques enhanced the radiation performance. A maximum gain of 12.2 dBi and a radiation efficiency of 86.6% were reported.

In short, antennas play an important role in detection and wireless communications. The performance of the THz system directly depends on the performance of the antenna [13]. Previous researchers have designed many THz antennas. They propose antennas that are either larger or have narrower bandwidths. Therefore, it is necessary to further study the antenna structure to meet the requirements of broadband and high gain. The array antenna has the advantages of high gain, directional radiation, wide-angle beam scanning, adaptive beamforming, multi-beam, and wide bandwidth. Due to all these advantages, the array antenna has two main problems: a high side-lobe level and grating lobe caused by improper element spacing. People have performed a lot of research on various types of array antennas. Ely Levine has proven that the gain and directivity of the microstrip array antenna are improved through empirical tests, but it is also obvious that with the increase in the number of radiation elements, the radiation loss, dielectric loss, and ohmic loss increase, while the surface wave loss remains unchanged [14]. In order to improve the antenna gain, a common practice is to place a dielectric lens, usually made of silicon, under the substrate, which is inspired by the use of THz technology in astrophysics [15]. However, for the application of the preferred graphic design, this remedy for enhanced substrate radiation becomes unavailable. A grounded coplanar waveguide (GCPW) is designed to control the radiation loss [16]. GCPW feed not only provides good impedance matching and sufficient substrate thickness, but also provides low transmission loss and small dispersion, and most importantly, it is easy to integrate with monolithic circuit design [17]. SIW technology reduces surface waves by reducing leakage waves and directing them in the desired direction. These are high-efficiency and low-loss antennas that can operate in higher TE modes and solve the problems of radiation loss and surface wave loss encountered in microstrip antennas [18]. SIW requires a high degree of precision milling, especially in THz band design, because the size and distance of the pass are becoming smaller and smaller. SIW antennas and microstrip feeders make the design compact and easy to implement [19]. By using the low-profile design of the Vivaldi antenna, significant bandwidth improvement can be achieved through a low side-lobe level (SLL) and mutual coupling [20]. The demand for high gain and wider bandwidth has influenced the study of various structural combinations, one of which is a magnetoelectric dipole with SIW microstrip patch elements [21].

In this paper, an antenna with high gain and broadband characteristics in the THz band is designed by using GCPW technology. This antenna has a unique structure, which is a three-layer design, and uses PTFE as a dielectric substrate. By adding metal through-holes into the dielectric layer, an SIW structure is formed to prevent electromagnetic energy leakage. By adding a fence isolation structure between the radiation units, the coupling effect between the radiation patches is significantly reduced, thereby improving the radiation bandwidth and gain of the antenna. In addition, the performance of the antenna under different parameter conditions is studied in depth, including the length of the fence isolation structure, the spacing distance of the radiation patch unit, the height of the dielectric substrate, the radius of the metal through-hole, and the thickness of the radiation patch. The structure of this paper is as follows: Section 2 explains the design of the THz high-gain antenna and the mathematical modeling of array structure. Section 3 optimizes the parameters of the designed antenna, and simulates and analyzes its radiation performance. Finally, Section 4 gives the conclusion of this paper.

## 2. Antenna Design

**Figure 1:**
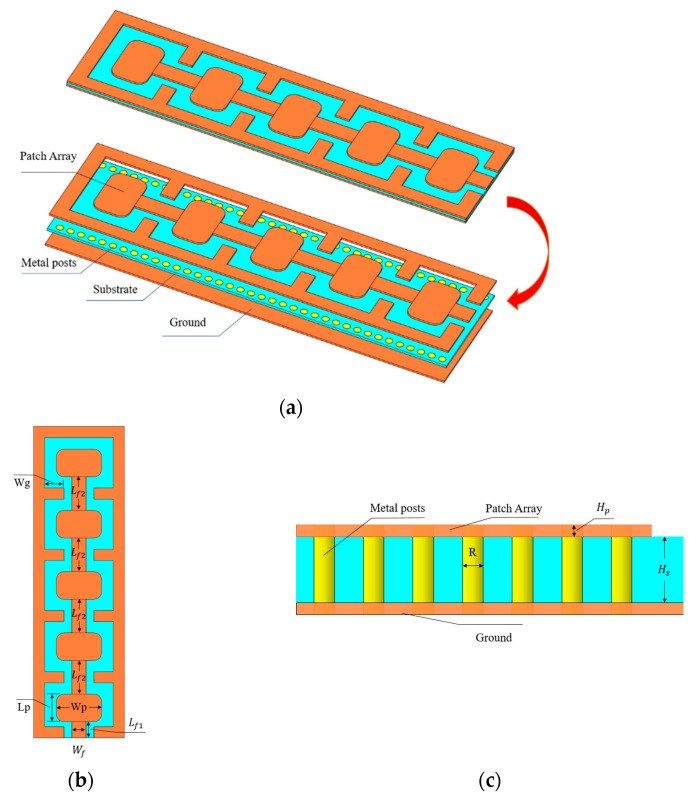
Antenna geometry. (**a**) Antenna basic structure decomposition. (**b**) Top view. (**c**) Side view.

Figure 1 is the antenna structure, showing the basic structure diagram, and top view and side view. The antenna is designed on a single-layer printed circuit board (PCB), which brings the advantages of a simple process and low integration and manufacturing cost. The antenna uses a PTFE material with a thickness of 26 μm as the dielectric substrate (the dielectric constant is 2.95, and the loss tangent is 0.0028). The array is composed of five symmetrical radiation patches. The patch shape is a rounded rectangle, and the antenna array is fed by GCPW. The GCPW is etched on the first copper layer, the second copper layer is the ground, and the radiation and grounding patches are made of 19-μm-thick copper (σ = 5.8 × 10^7^ S/m). The radiation patch has a certain periodic distance, and the antenna performance is degraded due to electromagnetic energy leakage and mutual coupling. Therefore, a metal cylindrical through-hole and fence structure are introduced to improve the radiation performance. The metal via makes the antenna have an SIW structure. The SIW technology can be integrated into the dielectric substrate and has the characteristics of low insertion loss and low radiation. It is realized by using a metalized via array on a low-loss dielectric substrate with a metal layer on the upper and lower bottom surfaces. The purpose is to realize the function of the traditional metal waveguide on the dielectric substrate. It can effectively realize passive and active integration, miniaturize the THz system, and greatly reduce the cost [22,23,24,25]. Moreover, its propagation characteristics are similar to those of rectangular metal waveguides, so the THz wave components and subsystems of SIW structure have the advantages of a high Q value, high power capacity, and easy integration. Since the entire structure is composed of a metallized through-hole array on a dielectric substrate, this structure can be accurately implemented using PCB or low-temperature co-fired ceramic (LTCC) processes and can be seamlessly integrated with microstrip circuits. Compared with the processing cost of traditional waveguide devices, the processing cost of SIW devices is very low and does not require any post-debugging work, which is suitable for integrated circuit design and mass production [26,27,28,29,30,31,32,33].

As shown in Figure 1b, both the base and the ground are rectangular. Five identical rounded rectangular antenna elements of size $L_{p}$ × $W_{p}$ on the upper surface of the substrate are arranged in a sequential configuration and connected by rectangular strips of size $L_{f2}$. The radiation patch is surrounded by a metal fence structure, which is connected to the ground through metal vias with a width of *Wg*. The radius of the metal through-hole is R, the length of the GCPW feeder is $L_{f1}$, and the width is $W_{f}$. Detailed dimensions are listed in Table 1.

The length $L_{p}$ and width $W_{p}$ of the radiant patch are calculated by the following formula:

(1) $$W_{p}=\frac{\left(2M+1\right)}{\sqrt{\varepsilon_{r}}}\times \frac{\lambda_{0}}{2}$$

(2) $$L_{p}=\frac{\left(2N+1\right)}{\sqrt{\varepsilon_{eff}}}\times \left(\frac{\lambda }{2}\right)-2\times ∆L$$

*M* and *N* are positive constants, both of which take the value 1 in this design. $\lambda_{0}$ is the free space wavelength, λ is the antenna operating wavelength, $\varepsilon_{r}$ is the relative dielectric constant of the medium, $\varepsilon_{eff}$ is the effective dielectric constant, and ∆L is the radiation patch length extension caused by the edge effect. $Z_{a}$ is the impedance of the series structure of five radiation patches, calculated according to the following formula:

(3) $$Z_{a}=\frac{11.96\lambda_{0}}{W_{p}}$$

$Z_{a}$ is standard 50 Ω impedance matching, $Z_{0}$ is normalized impedance, and the transmission line size computation formula is as follows:

(4) $$Z_{0}=\sqrt[{}]{50\times Z_{a}}$$

(5) $$W_{f}=\frac{7.457H_{s}}{e^{\frac{Z_{0}\sqrt{\varepsilon_{r}+1.41}}{87}}}-1.25H_{p}$$

(6) $$L_{f1}=(2P+1)\times \frac{\lambda }{4}$$

(7) $$L_{f2}=\left(2Q+1\right)\times \frac{\lambda }{2}+2\times ∆L$$

**Figure 2:**
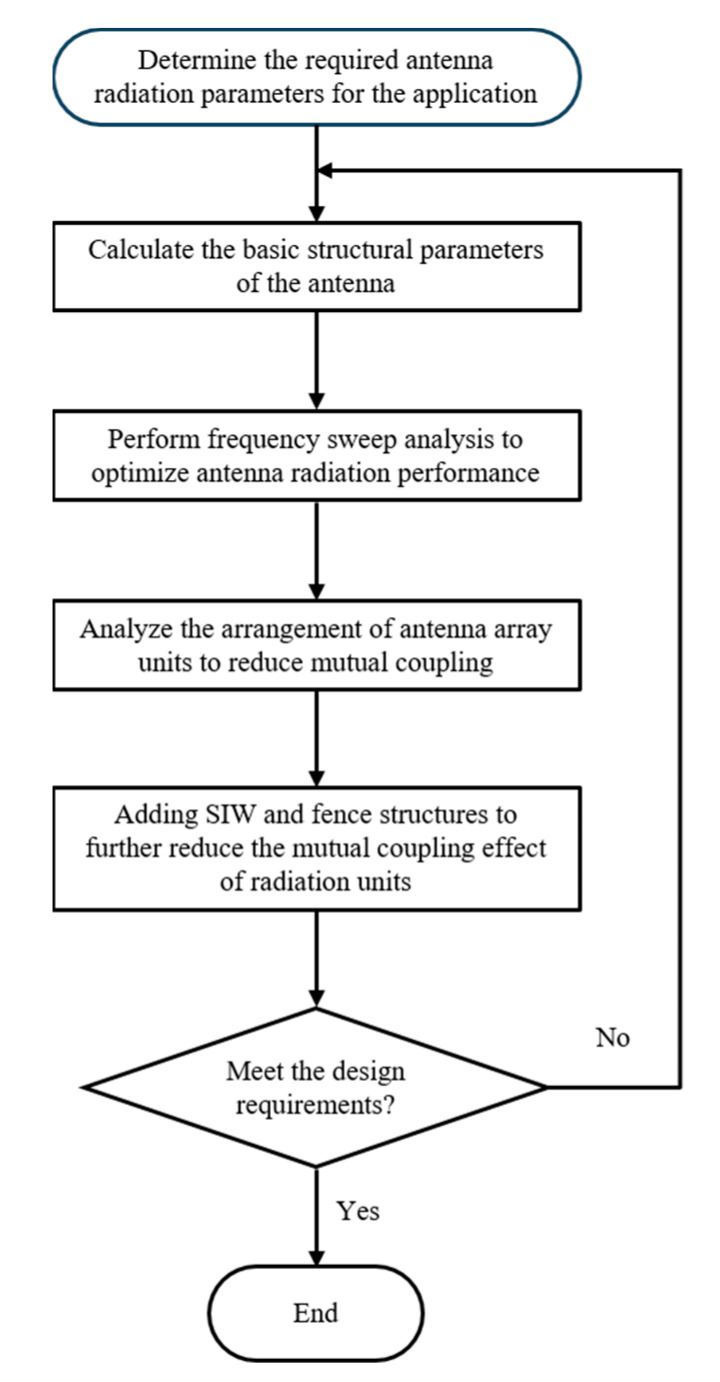
Antenna design process.

$H_{s}$ is the thickness of the dielectric layer, $H_{p}$ is the thickness of the radiating layer copper plate, *P* and *Q* are positive integers, and the values of *P* and *Q* in this design are 1. The antenna size is optimized by using computer simulation technology software (CST Studio Suite 2023). Figure 2 is the flow chart of the proposed antenna design method. In order to improve the antenna gain and frequency bandwidth, the antenna design steps are as follows:

Step 1: Determine the antenna radiation parameters and size required in the product structure;

Step 2: Carry out relevant theoretical calculation to obtain the basic structure, material medium, and size parameters of the antenna;

Step 3: Perform a sweep frequency analysis on the relevant results to optimize the antenna radiation performance;

Step 4: Analyze the antenna array structure, including arrangement layout, feed connection mode, unit spacing, etc., to reduce the coupling between each other and enhance the radiation performance;

Step 5: Add SIW and fence structure, adjust the radius of the through-hole and the width of the partition patch, and further reduce the mutual coupling of radiation units;

Step 6: If the relevant results cannot meet the application requirements after optimization, return to the first step to redesign.

## 3. Antenna Performance Analysis

THz antennas play an important role in the high data rate transmission of wireless communication systems. The CST Microwave Studio is used as a simulation tool to analyze antenna radiation properties in the frequency range of 0.6–0.7 THz, including the gain, return loss (S11) and voltage standing wave ratio (VSWR), bandwidth, and radiation mode efficiency.

**Figure 3:**
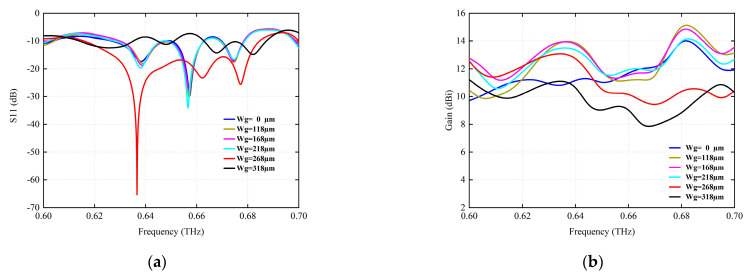
The simulation of the variation of antenna radiation performance for different fence isolation lengths *Wg* (µm). (**a**) S11 (dB). (**b**) Gain (dBi).

By introducing fence structure to optimize antenna radiation performance, the highest bandwidth and excellent effect of antenna design are obtained. Inserting the fence structure between the antenna radiation elements can enhance the isolation effect between the antenna elements and reduce the coupling effect between the elements. The fence length *Wg* is varied from 0 µm to 318 µm to analyze its effect on the radiation performance. As shown in Figure 3a, when *Wg* is 0 µm, there is no fence isolation structure between antenna units, and the antenna has three resonant points of 0.6381 THz, 0.6573 THz, and 0.6746 THz. The range of return loss less than −10 dB is 0.6265–0.6478 THz, 0.6499–0.6628 THz, and 0.6698–0.6785 THz. At 0.6573 THz, the antenna return loss is −29.72 dB, and the antenna bandwidth is low due to the coupling between the radiating elements. When the length of the barrier isolation structure *Wg* increases, the bandwidth performance of the antenna improves, and the optimal radiation bandwidth of the antenna is obtained when the value of *Wg* is 268 µm.

**Figure 4:**
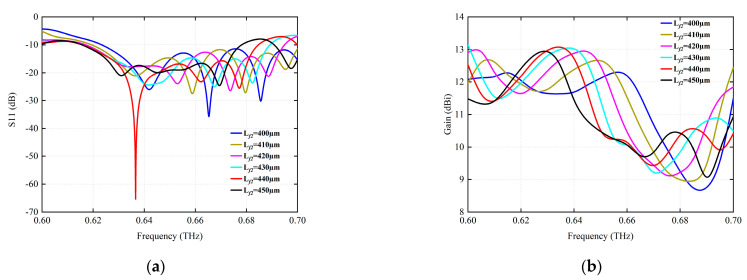
The simulation of the influence of different unit interval distances $L_{f2}$ (µm) on radiation characteristics. (**a**) S11 (dB). (**b**) Gain (dBi).

Each antenna radiation unit is arranged in a row sequence. The separation distance between the radiating elements plays a very important role in the operating frequency band and the radiating performance, as they affect the antenna array in the return loss. For comparison, the effects of different unit spacing distances on antenna return loss and radiation gain are shown in Figure 4. The interval distance analysis considers 400–450 µm, as shown in Figure 4a, where a separation distance of 440 µm provides a better return loss with the highest gain around its resonant frequency point.

**Figure 5:**
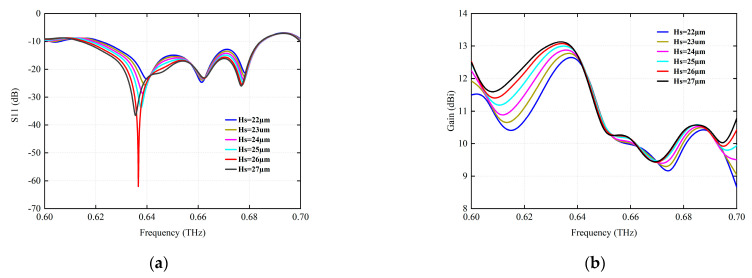
The simulation of the radiation parameters at different media thicknesses $H_{s}$ (µm). (**a**) S11 (dB). (**b**) Gain (dBi).

Figure 5 analyzes the influence of different dielectric thickness $H_{s}$ on the radiation performance. Obviously, with the increase in dielectric thickness, the designed resonant frequency moves from high frequency to low frequency, which makes it easy to excite surface waves, increase surface loss, and reduce antenna gain and efficiency. It can be seen from Figure 5a that the antenna return loss is optimal when $H_{s}$ is 26 µm, which is represented by the red solid line.

**Figure 6:**
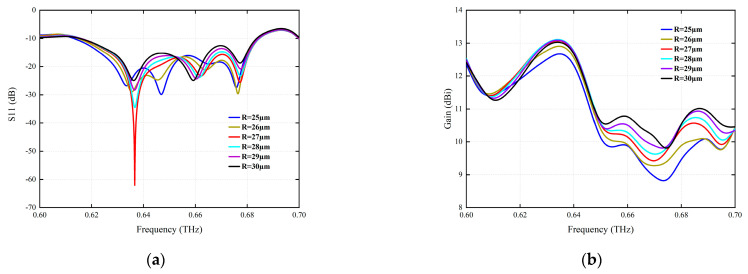
The simulation of the radiation parameters for different via radius R (µm). (**a**) S11 (dB). (**b**) Gain (dBi).

Similarly, when the via radius R is increased, the variation of the antenna characteristics in the range of a 25-to-30-µm via radius is investigated, as shown in Figure 6. For the SIW structure, the influence of the through-hole radius on the return loss and observed gain is analyzed. As shown in Figure 6a, it can be clearly seen that the value of the reflux loss factor with the optimal resonant frequency is obtained at a radius of 27 μm.

**Figure 7:**
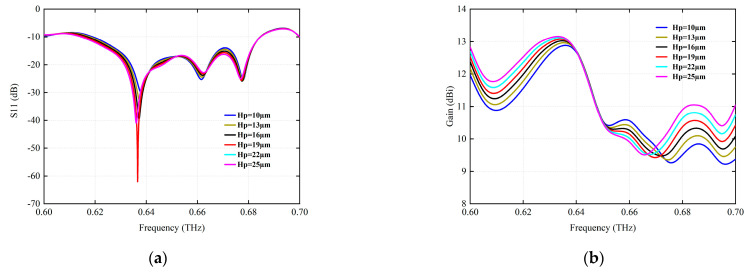
The simulation of the radiation parameters under different radiation patch thickness $H_{p}$ (μm). (**a**) S11 (dB). (**b**) Gain (dBi).

Figure 7a shows the effect of different radiation patch thickness $H_{p}$ on the backflow loss. The results show that the return loss varies slightly with the thickness of the patch. Similarly, Figure 7b shows a change in radiation gain, and it is found that the observed change is very slight. Therefore, it can be considered that the patch antenna with a patch thickness of 27 μm has better performance at a resonance frequency of 0.6366 THz. In addition, the performance of the antenna does not represent too much change with the thickness of the radiation patch, except for the return loss factor.

**Figure 8:**
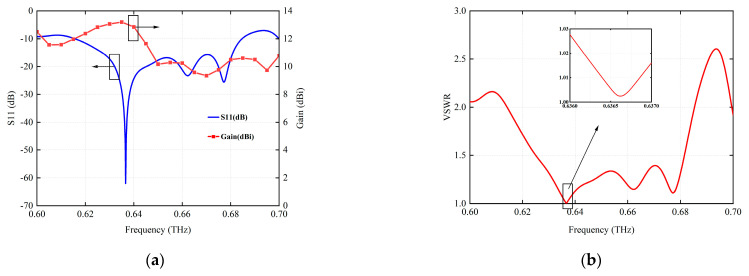
Radiation characteristics of the antenna after final optimization. (**a**) S11 and gain. (**b**) VSWR.

Figure 8 shows the simulation results of the final optimized antenna radiation characteristics. Figure 8a shows that the resonant frequency of the antenna is 0.6366 THz, and the corresponding return loss is −62 dB. In this case, the radiation gain is 13.05 dBi and the bandwidth is 0.61–0.68 THz. The antenna peak gain is 13.14 dBi, corresponding frequency is 0.6350 THz, and return loss is −28.84 dB. As can be seen in Figure 8b, the VSWR of the antenna is less than 2.0 in most ranges of the 0.6–0.7 THz frequency band, and the VSWR reaches 1.00158 when the center frequency is 0.6366 THz. Ideally, the VSWR should equal 1, indicating that the antenna receives 100% of the power and zero reflection [34]. The results show that the design structure adopted in this paper reduces the VSWR of the antenna.

**Figure 9:**
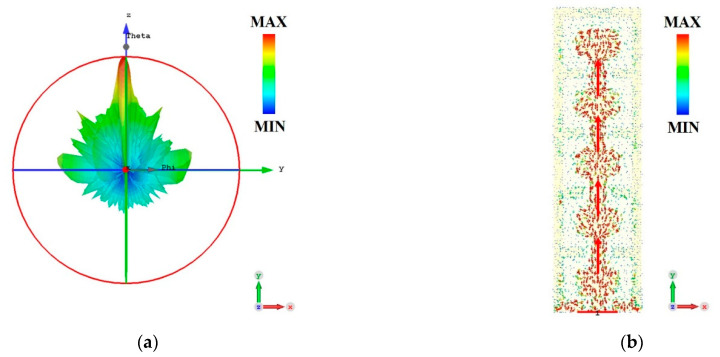
Simulation results of antenna at center frequency 0.6366 THz, and the red arrows indicate the direction in which the currents converge. (**a**) Three-dimensional FRP. (**b**) Antenna surface current distribution.

Figure 9a simulates the 3D far-field radiation pattern (FRP) of the antenna at the center frequency of 0.6366 THz, and Figure 9b shows the antenna surface current distribution at this frequency. Electromagnetic energy (including radiated E-field energy and H-field energy) is transmitted along the GCPW, effectively transporting the energy from the connection lines to the radiation patch, and radiated by the radiation patch.

**Figure 10:**
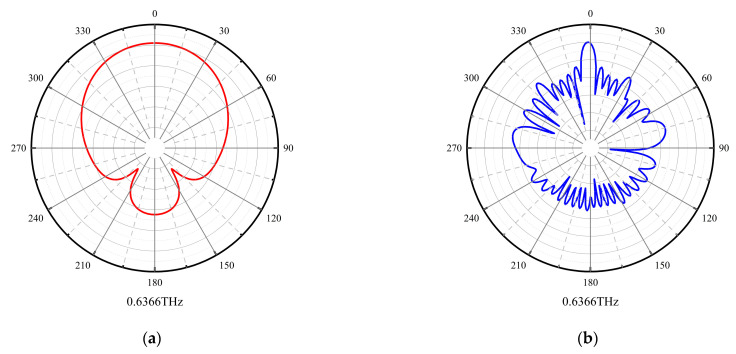

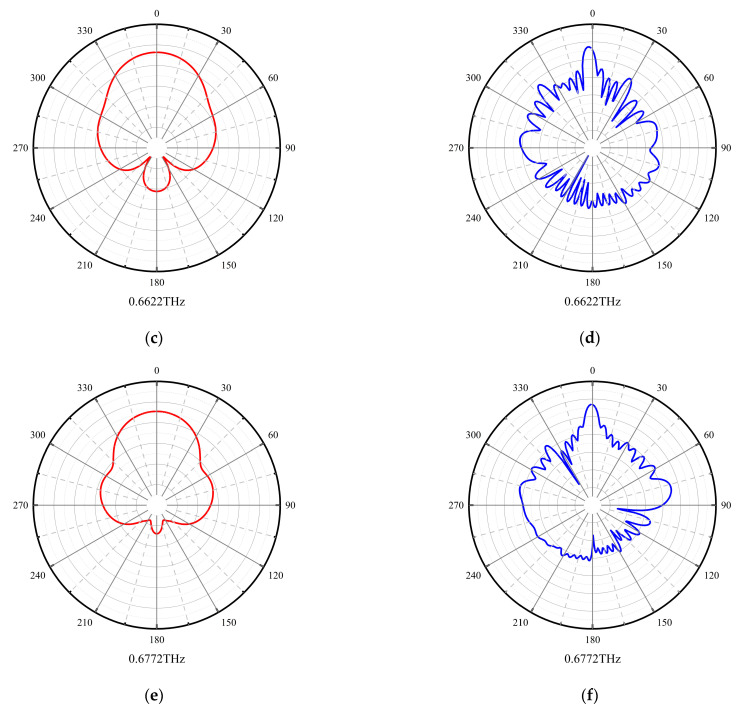
Radiation direction of the antenna, the red line is the XOZ plane and the blue line is YOZ plane. (**a**) XOZ plane at 0.6366 THz. (**b**) YOZ plane at 0.6336 THz. (**c**) XOZ plane at 0.6622 THz. (**d**) YOZ plane at 0.6622 THz. (**e**) XOZ plane at 0.6772 THz. (**f**) YOZ plane at 0.6772 THz.

Figure 10 shows the radiation pattern of the antenna at 0.6366 THz, 0.6622 THz, and 0.6772 THz; Figure 10a,c,e is the XOZ plane, and Figure 10b,d,f is the YOZ plane.

As shown in Table 2, the designed antenna is compared with the related THz high-gain antennas in recent years. The Photonic Band Gap (PBG) structure is adopted in [35,36,37]. The curvature radius of PBG affects the gain and directivity of the antenna. In Reference [38], a new microstrip patch antenna array with a silicon–air photonic crystal substrate was formed by adding a through-hole structure to a uniform silicon substrate, and its performance was analyzed in the frequency range of 0.58~0.72 THz by suppressing the bad surface wave in the uniform substrate. It shows that the PBG substrate can effectively improve the characteristics of the traditional microstrip antenna array, and obtain the return loss, bandwidth, gain, and radiation efficiency enhancement. The design of a reconfigurable radiation antenna proposed in [39] is based on the integration of a reconfigurable fractal antenna and electro-optic substrate material. The electromagnetic characteristics of a fractal antenna are controlled at the level of the fractal geometry, electric length, and dielectric substrate, and have a multi-band response. The change in geometry and length produces a large frequency shift, while the dielectric change in polymer-dispersed liquid crystal (PDLC) produces fine and continuous tuning, but the relative bandwidth is narrow and the gain is not high. In order to improve the performance parameters of the antenna, a feasible method based on the concepts of metamaterial (MTM) and SIW is adopted in [40]. The tapered slot on the radiation patch and the metal through-hole at the edge of the structure are realized without increasing the physical size of the structure. By comparing with the antenna bandwidth, radiation gain, directivity, return loss, standing wave ratio, and other parameters in the above literature, the antenna in this paper has better bandwidth and radiation gain.

## 4. Conclusions

In this paper, a high-gain THz antenna based on the SIW structure of a new microstrip patch antenna and PCB technology is studied. The antenna uses PTFE material as the dielectric substrate, and the upper and lower sides are, respectively, copper-plated as the radiation surface and the ground, and a GCPW structure is used as the feed unit. By adjusting the thickness of the dielectric layer, the size of the radiation patch, and the size of the through-hole radius, the radiation parameters of the antenna are optimized and higher antenna gain is obtained. With the increase in the substrate height, the resonant frequency of the antenna shifts to low frequency, and the gain decreases significantly. The peak gain of the simulated antenna is 13.14 dBi. In the frequency range from 0.61 THz to 0.68 THz, the return loss of the antenna is better than −10 dB. At the corresponding resonant frequency of 0.6366 THz, the gain and directivity of the antenna are 13.05 dBi and 15.1 dBi, respectively, and the minimum return loss of the simulated antenna is −62 dB. The antenna has the advantages of a simple feeder structure, low manufacturing cost, wide bandwidth, and high gain, and is suitable for THz high-speed-communication integrated antenna front-end and system applications.

## Figures and Tables

**Table 1:** Design dimensions of THz antenna array.

Parameters	Values (µm)	Detailed Description
$L_{p}$	360.9	Radiation patch length
$W_{p}$	590.4	Radiation patch width
$L_{f1}$	220	GCPW feeder length
$L_{f2}$	440	Radiation unit connection line length
$W_{f}$	180	Connecting line width
$H_{s}$	26	Dielectric substrate thickness
$H_{p}$	19	Radiation patch thickness
*R*	27	Metal through-hole radius
*Wg*	268	Fence isolation structure length

**Table 2:** Comparison Results.

Ref.	Bandwidth (THz)	Antenna Structure	Resonant Frequency (THz)	Gain (dBi)	Directivity (dBi)	S11 (dB)	VSWR
[35]	0.678–0.702	PBG	0.690	6.793	6.914	−64.16	1.00124
[36]	0.594–0.702	PBG	0.601	11.60	12.21	−53.66	1.00416
[37]	0.663–0.683	PBG	0.670	6.32	7.09	−26.27	1.10213
[38]	0.60–0.63	PBG	0.607	9.75	-	−67.2	1.00087
[39]	0.504–0.540	PDLC	0.522	4.92	8.43	−14.5	1.46416
[40]	0.600–0.622	SIW MTM	0.617	1.8	-	−34	1.04072
This Work	0.61–0.68	GCPW SIW	0.6366	13.05	15.1	−62	1.00158

## Data Availability

The data that support the findings of this study are available from the corresponding author upon reasonable request.
